# Biocompatibility and Biodegradation Studies of Subconjunctival Implants in Rabbit Eyes

**DOI:** 10.1371/journal.pone.0022507

**Published:** 2011-07-22

**Authors:** Yan Peng, Marcus Ang, Selin Foo, Wing Sum Lee, Zhen Ma, Subbu S. Venkatraman, Tina T. Wong

**Affiliations:** 1 School of Materials Science and Engineering, Nanyang Technological University, Singapore, Singapore; 2 Singapore National Eye Centre, Singapore, Singapore; 3 Singapore Eye Research Institute, Singapore, Singapore; 4 Department of Ophthalmology, Yong Yoo Lin School of Medicine, National University of Singapore, Singapore, Singapore; Consejo Superior de Investigaciones Cientificas, Spain

## Abstract

Sustained ocular drug delivery is difficult to achieve. Most drugs have poor penetration due to the multiple physiological barriers of the eye and are rapidly cleared if applied topically. Biodegradable subconjunctival implants with controlled drug release may circumvent these two problems. In our study, two microfilms (poly [d,l-lactide-co-glycolide] PLGA and poly[d,l-lactide-co-caprolactone] PLC were developed and evaluated for their degradation behavior in vitro and in vivo. We also evaluated the biocompatibility of both microfilms. Eighteen eyes (9 rabbits) were surgically implanted with one type of microfilm in each eye. Serial anterior-segment optical coherence tomography (AS-OCT) scans together with serial slit-lamp microscopy allowed us to measure thickness and cross-sectional area of the microfilms. In vitro studies revealed bulk degradation kinetics for both microfilms, while in vivo studies demonstrated surface erosion kinetics. Serial slit-lamp microscopy revealed no significant inflammation or vascularization in both types of implants (mean increase in vascularity grade PLGA50/50 12±0.5% vs. PLC70/30 15±0.6%; P = 0.91) over a period of 6 months. Histology, immunohistochemistry and immuno-fluorescence also revealed no significant inflammatory reaction from either of the microfilms, which confirmed that both microfilms are biocompatible. The duration of the drug delivery can be tailored by selecting the materials, which have different degradation kinetics, to suit the desired clinical therapeutic application.

## Introduction

The eye is the vital organ for sight and unique because it is both anatomically and immunologically privileged. While the eye is protected physiologically, it is also resistant to penetration by drugs. Topical application is the mainstay of most ocular therapy, but ocular bioavailability is poor due to the efficient protective barriers [1], [2], [3]. The recognition of this limitation in efficient ocular drug delivery has led to a range of systems that vary in mode of administration, implantation site, composition and vehicles [4], [5], [6], [7], [8], [9], [10], [11], [12], which all aim to circumvent the problems of drug bioavailability, sustainability and feasibility of administration [13].

Biodegradable polymers are proven vehicles for sustained drug delivery [5]. Polyhydroxyesters are easily fabricated with predictable biodegradation kinetics and biocompatible degradation [14]. These polymers, such as poly [d, l-lactide-co-glycolide] (PLGA) or poly [d, l-lactide-co-caprolactone] (PLC), degrade through hydrolysis of their ester bonds into lactic acids, glycolic acids and caproic acid – and eventually into water and carbon dioxide [15], [16], [17]. Since the body effectively deals with these degradation monomers, there is very minimal systemic toxicity associated with its use in human tissues. As such, these polymers have been used in US FDA-approved implantable devices or injectable pharmaceutical products.

PLGA has been used extensively in studies to deliver a wide variety of drugs using various forms such as microparticles, emulsions, implants and hydrogels [11], [12], [18], [19], [20]. The ability to tailor the polymer degradation time by altering the ratio of the monomers used during synthesis has made PLGA a common choice in the production of a variety of biomedical devices such as grafts, sutures, implants, root-canal fillings and prosthetic devices [5]. In comparison, the copolymer PLC is relatively new, and is currently finding application in implantable systems such as occluders for atrial septal defects [21], [22], [23]. Several studies have reported the development of biodegradable polymer microfilms specifically for ocular drug delivery [11], [21], [22]. Apart from the known advantages of using these biodegradable polymers, the ability to cast the microfilms with varied thickness ranging from microns to millimeters, is particularly useful for ease of insertion into various layers of the eye [24]. Moreover, these biodegradable microfilms may enable the release of multiple drugs with directionality and different release rates. Our authors have published significant results from preliminary *in vitro* studies using commonly used ophthalmic drugs, latanoprost and 5-fluorouracil [25].

In this study, we aim to develop and compare two different biodegradable microfilms for their potential application as vehicles for intraocular drug delivery, specifically on surgical insertion into the subconjunctival space. We evaluated the degradation behavior of both microfilms *in vitro* and *in vivo*, surgical feasibility and biocompatibility of both types of microfilms using three complementary techniques: slit-lamp microscopy, anterior segment optical coherence tomography (AS-OCT) and histology with various staining methods.

## Materials and Methods

### Materials

Polymers used in this study are poly (d,l-lactide-co-glycolide), PLGA50/50 (intrinsic viscosity 1.02 dl/g, Mw = 160 kDa) and poly (d,l-lactide-co-ε-caprolactone) PLC70/30 (intrinsic viscosity 1.66 dl/g, Mw = 210 kDa), which were purchased from Purac Far East Pte. Ltd., Singapore. High-performance liquid chromatography (HPLC)-grade dichloromethane and chloroform were from Tedia Company. Phosphate buffer saline (PBS) tablets were obtained from CalBioChem, England.

### Sample preparation

Samples of PLGA50/50 and PLC70/30 were weighed before dissolving the appropriate amount in dichloromethane. Following dissolution, the samples were dried in petri-dishes under a fume hood for a day, followed by drying in a vacuum oven at 37°C until the solvent level was less than 1% of the total weight, as measured using a thermo-gravimetric analyzer (TGA, TA instruments Q500). After drying, all samples were cut manually into standard sized microfilms (6.0×3.0×0.5 mm) by using a sharp knife.

### In vitro degradation study

Samples were immersed in a closed vial containing 5 ml Phosphate Buffered Saline (PBS, pH 7.4). PBS was prepared by dissolving PBS tablets into 1 liter deionized water. All vials were incubated at 37°C throughout the study. The buffer was refreshed every week, and at every predetermined time point, samples were taken out, rinsed with deionized water and dried in 37°C vacuum oven for 7 days, before testing. Degradation of PLGA50/50 and PLC 70/30 was monitored by film thickness (measured by Elcometer 456), water absorption, weight loss and weight average molecular mass (Mw) and poly dispersity index(PDI). Dried samples were dissolved in chloroform (1–5 mg/ml) and filtered through 0.22 µm regenerated cellulose syringe driven filters before test. Weight average molar mass and poly dispersity of the sample were determined by gel permeation chromatography (GPC, Agilent 1100) at 35°C, using Agilent PLgel 5 µm mixed-C column, under a flow rate of 1 ml chloroform per minute, using a Refractive Index Detector (RID).

### Sterilization

All the samples were sterilized by ethylene oxide (ETO) at 37°C (used for normal medical device) in Tan Tock Seng Hospital (Singapore) prior insertion into animals.

### Surgical insertion of microfilms

We obtained approval from the SingHealth Institute Animal Care and Use Committee (IACUC Singhealth Approval Number 2009/SHS/478) and all procedures were performed in accordance with the ARVO Statement for the Use of Animals in Ophthalmic and Vision Research. Nine New Zealand white rabbits (18 eyes) were used aged 4–6 months old with a weight range of 2–2.5 kg each. Each rabbit was anesthetized with intraperitoneal injection of ketamine hydrochloride (35–50 mg/kg) and Xylazil (5–10 mg/kg). After the animal had been adequately anaesthetized, the eye was cleaned with povidone–iodine (10%) and draped with sterile cloth. A subconjunctival pocket was created via blunt dissection just at the limbus with a 5–6 mm incision in the superior-temporal aspect of the rabbit's eye. Microfilms were sterilized in ethyl alcohol and chlorhexidine before soaking in sterile normal saline. The microfilm was then inserted into the subconjunctival pocket 1 mm from the limbus using a conjunctival forceps. Closure with 10-0 nylon sutures was done to ensure secure implantation of each microfilm. In each rabbit, PLC70/30 (n = 9) microfilms were inserted into the right eye, whilst PLGA50/50 (n = 9) microfilms were inserted into the left eye. Topical Tobradex (Tobramycin & Dexamethasone) was administered each eye 4 times a day for 5 days.

### Clinical monitoring

Visual inspection of the operated eyes was conducted daily following surgery. The animals' eyes were also inspected for changes at the insertion site, gross appearance of the implant and for any evidence of infection. Slit-lamp examination of the exterior and anterior chamber of the eyes was done prior to surgery and weekly thereafter. All clinical and ocular observations were recorded on a chart. The test animals were also monitored for any gross changes such as eye discharge, squinting and, ocular discomfort. A modified Hackett McDonald ocular score was used to record the presence of conjunctival injection, swelling, discharge and corneal clarity [26]. Two masked independent investigators (MA, TTW) objectively graded each eye based on slit lamp photography.

### Anterior Segment Optical Coherence Tomography

Anterior segment photographs and anterior segment optical coherence tomography (AS-OCT, Visante OCT, Carl Zeiss Meditec Inc., Germany) of the implanted eyes was performed at monthly intervals. The Visante OCT is a high-resolution biomicroscopic device for anterior segment imaging (axial resolution = 18 mm), based the principle of low coherence interferometry using a 1310 nm light emitting diode [27]. Due to the optical properties of different tissues, the AS-OCT image can help us identify internal structures of the eye, such as fluid, scarring or thinning of the sclera or conjunctiva [28]. We used a modified technique previously described to obtain standardized images of the implanted microfilm in each rabbit eye by a single, masked operator (WSL) [29]. A radial anterior segment line scan was chosen to include both the implanted microfilm and the surgical insertion site. The site of conjunctival elevation from the microfilm was determined by the location of a light reflex over the conjunctiva during image acquisition. In cases where the light reflex was absent, the observer manually assessed the surface of the microfilm to select a radius that contained elevation. This AS-OCT technique allowed us to image the layers of the eye, location of implant as well as obtain standardized measurements of the implant, which was included microfilm thickness and length. The Anterior Segment OCT (AS-OCT) is calibrated internally to detect internal structures of the eye using high-resolution corneal and angle scans and pachymetry maps at a rate of up to 2048 A-scans per second, with an optical axial resolution of up to 18 mm and optical transverse resolution of up to 60 mm (Carl Zeiss Meditec Inc, www.meditec.zeiss.com).

### Enucleation and preparation of tissue and microfilms

Three rabbits were randomly selected for euthanasia at 1, 3 and 6 months post-implantation. Euthanasia was carried out with intraperitoneal pentobarbitone (60–150 mg/kg) followed by enucleation of both eyes. The eyes were immediately immersed in a mixture of 4% glutaraldehyde and 2.5% neutral buffered formalin for 24 hours. The globes were dehydrated and embedded in paraffin, then sent to the histopathological laboratory for sectioning with a microtome, appropriate staining (Haematoxylin and Eosin and Masson Trichrome stain) and reading – for signs of inflammation, tissue damage, scarring and fibrosis. Immunohistochemistry stains were used to quantify the amount of encapsulation that developed in each eye.

### Immunohistochemistry

The sectioned, paraffin-embedded slides were heated to 60°C for 1 hour, deparaffinized, and rehydrated. Endogenous peroxidase activity was quenched by a 30-minute incubation in 3% H_2_O_2_/PBS solution, washed, and blocked with 20% Aquablock (East Coast Biologics, Inc., North Berwick, ME) in PBS/0.2% Tween-20 for 30 minutes. Sections were incubated with a rabbit anti-mouse CD45 monoclonal antibody (mAb) at 10 µg/ml (F4/80, Serotec, Raleigh, NC; CD45, BD Biosciences Pharmingen, San Diego, CA). Detection of the primary antibodies was performed with biotinylated rabbit anti-rabbit IgG secondary antibodies (2 µg/ml, Vector Laboratories, Orton Southgate, UK) followed by incubation with 3,3′-diaminobenzidine (DAB substrate kit; Vector Laboratories, Burlingame, CA) and counterstained with Hematoxylin QS (Vector Laboratories).

### Immunofluoresence

The sections were de-paraffinized with xylene and rehydrated. Antigen retrieval was performed before heating the sections to 100°C for 25 minutes. The sections were cooled and washed with PBS and 1%BSA for 30 minutes before incubating in a dark incubation chamber for 90 mins with 50 ul of primary antibody at room temperature. Images of the sections were then viewed and digitally captured with a fluorescent microscope.

### Histological analysis

The amount of fibrosis and scarring was evaluated by measuring the thickness of the collagen capsule formed around each implant. The sections were stained with Masson Trichrome, and the average thickness of the collagen capsule was measured by integration of the collagen-stained area throughout the entire length of the implant. Next, the total inflammatory cell response was estimated by determining the percentage of the implant surface lined by inflammatory cells. We calculated the ratio L: C [L = total length of the implant surface and C = length of implant surface lined by inflammatory cells (CD45 stained) immediately adjacent to the implant surface]. In sections where the implant was not clearly visible, the implant site was sectioned and stained for inflammatory cells.

### Statistical analysis

Statistical analysis included descriptive statistics, where the mean and standard deviation (SD) was calculated for the continuous variables; while frequency distribution and percentages were used for categorical variables. The Student's t-test was used to analyze immunohistochemistry data and independent samples t-test was used to study the rate of degradation of the microfilms. P-values of less than 0.05 were defined as statistically significant. All data was expressed as mean ± SD unless otherwise stated. All analyses were performed using STATA version 11 (StataCorp LP, College Station, Texas, USA).

## Results

### In vitro degradation study

We studied various factors *in vitro*, such as water absorption, weight loss, change in thickness and change in molar mass, to analyze the degradation of both types of microfilms.

#### Water absorption

**Figure 1:**
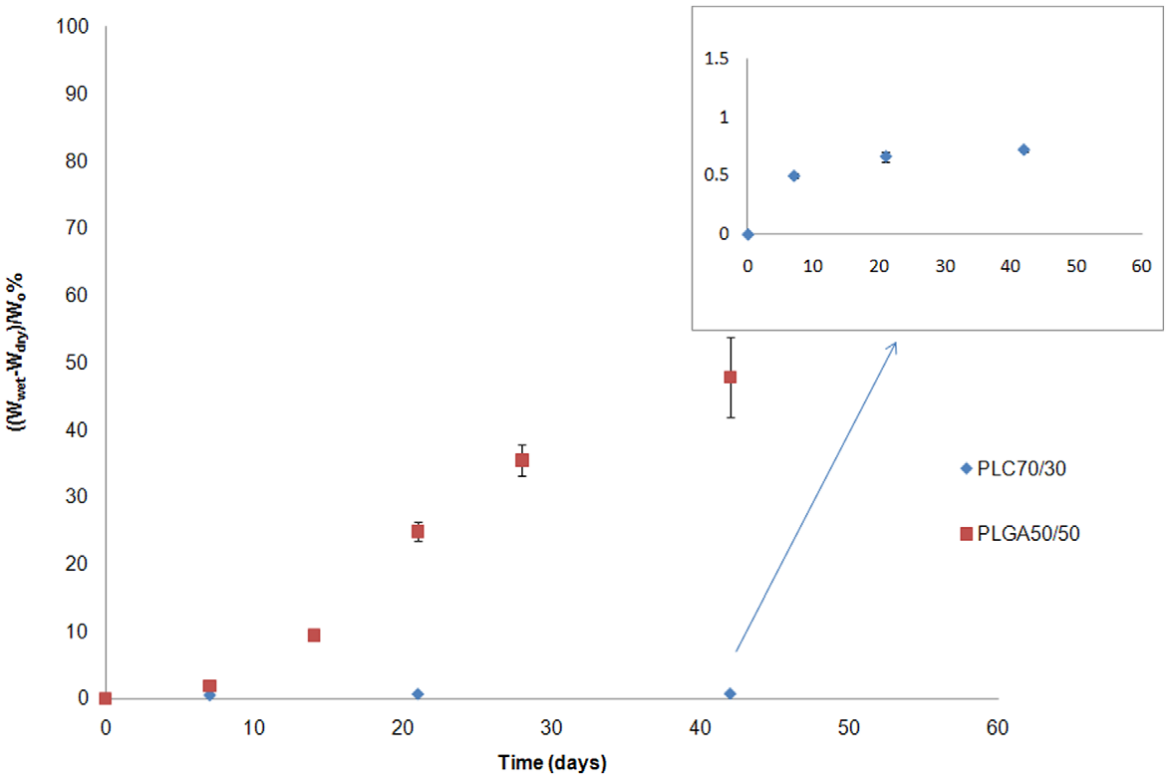
In vitro water absorption of PLGA50/50 and PLC70/30, immersed in PBS buffer (pH 7.4).

As these polymers degrade in the body by simple hydrolysis, water absorption rates are indicative of hydrolysis rates. The amount of water absorbed by the sample was calculated as: Water absorption = (W_wet_−W_dry_)/W_o_ %, where W_wet_ represents the weight of the wet sample after wiping by tissue, W_dry_ represent the final weight of the dried samples, and W_o_ represent the sample's initial weight. The amount of water absorbed in the microfilm increased with immersion time for both polymers (Figure 1). PLGA started to absorb significant amounts of water after a week, whereas PLC 70/30 absorbed only about 1% of water over 6 weeks. This was primarily due to two factors: first, the PLGA 50/50 is completely amorphous with a T_g_ close to 40°C, and this allows quicker water penetration. The PLC 70/30 is semi-crystalline, and water absorption is limited to the amorphous phase. Second, the glycolide segment is more hydrophilic than the lactide moiety, which results in a more hydrophilic PLGA 50/50 matrix. Moreover, once significant water absorption occurs, the hydrolysis is accelerated, resulting in oligomers that are increasingly more water-soluble.

#### Mass loss

**Figure 2:**
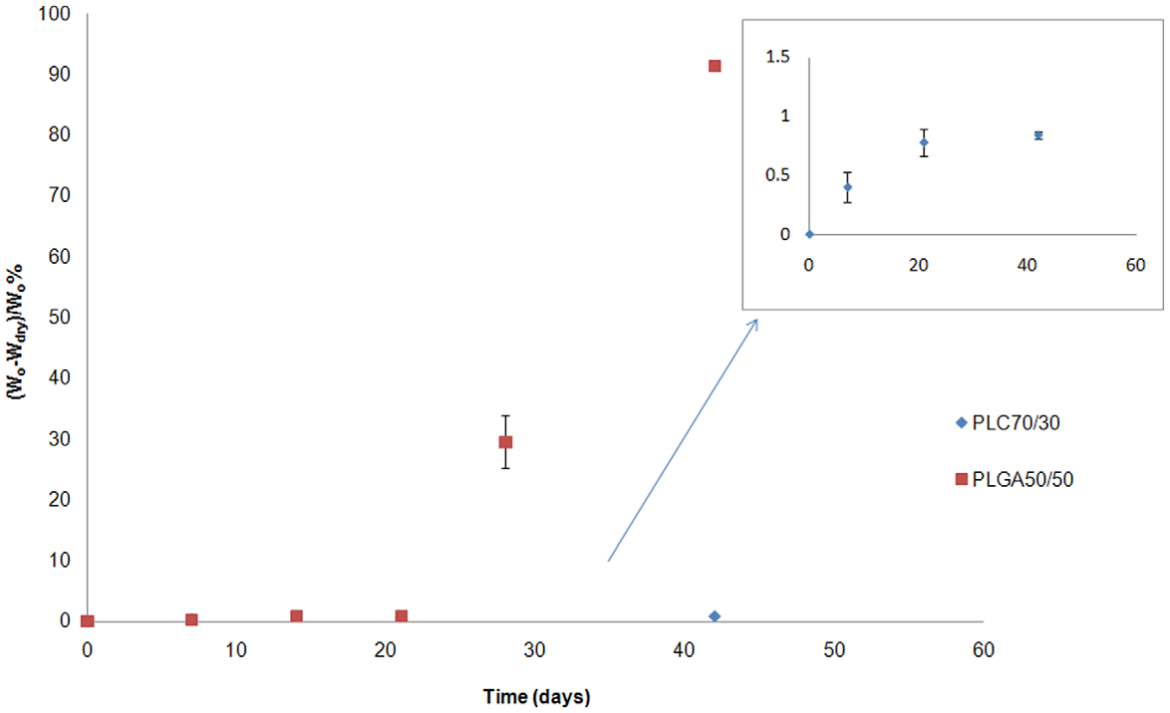
In vitro weight loss of PLGA50/50 and PLC70/30, immersed in PBS buffer (pH 7.4).

Mass loss was calculated as: (W_o_−W_dry_)/W_o_ %. In conjunction with water absorption, mass loss started earlier for PLGA 50/50. At 3 weeks, PLGA 50/50 started to lose significant mass, whereas PLC 70/30 did not demonstrate any notable mass loss until after day 56 (Figure 2). As water absorption increased, PLGA 50/50 started to degrade, with oligomers being produced with carboxylic end groups (-COOH). Such oligomers become increasingly water-soluble as molar mass decreases. In contrast, PLC had absorbed very little water and hence the observed hydrolysis rate was low, with water-soluble oligomers not forming to any measurable extent until day 56.

#### Change of molecular weight (Mw) and PDI

**Figure 3:**
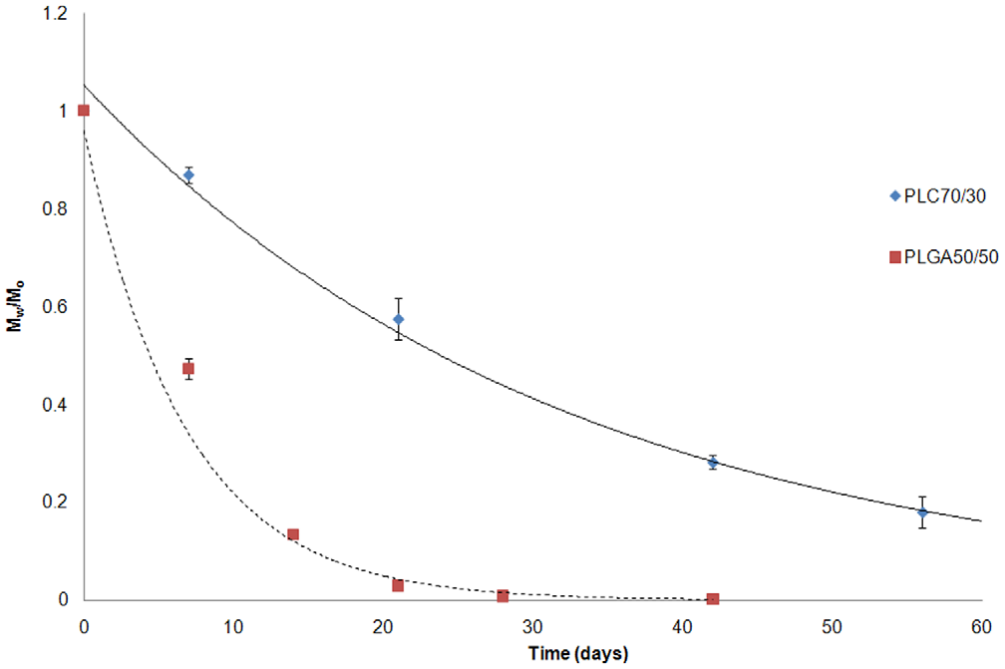
Monitoring of weight molecular mass for PLGA50/50 and PLC70/30 in vitro (PBS, pH 7.4).

There was a notable decrease in weight molecular mass for both PLGA50/50 and PLC70/30 over 56 days (Figure 3). The M_w_/M_o_ versus time graph showed that both polymers demonstrated bulk degradation with a thickness of 0.5 mm. PLGA50/50 degraded faster than PLC70/30, and the drop in M_w_ agreed with the corresponding water absorption and mass loss in these two materials.

**Figure 4:**
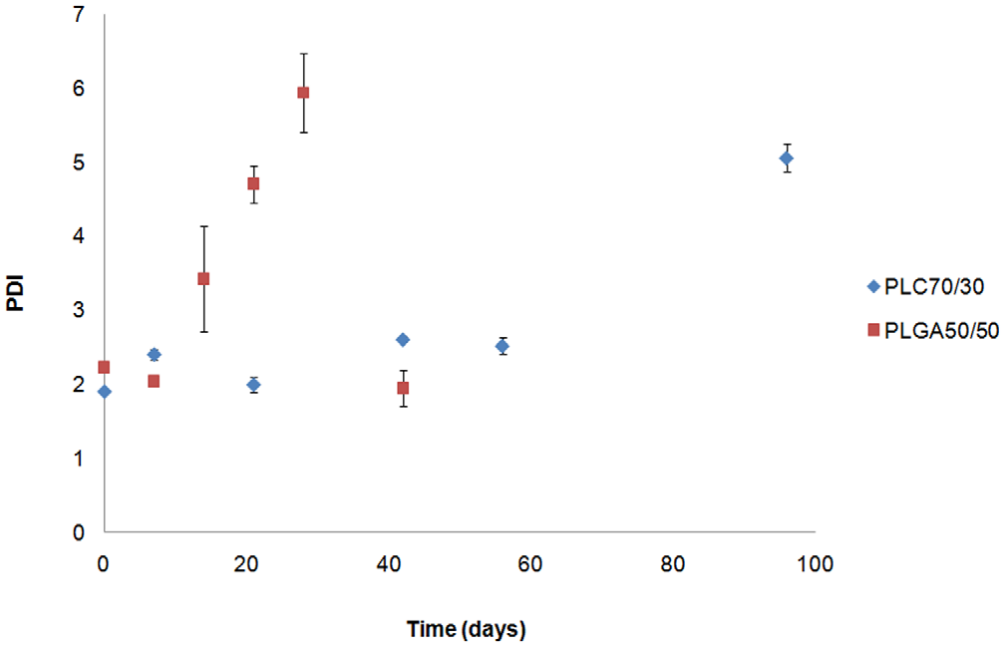
Monitoring of PDI for PLGA50/50 and PLC70/30 in vitro (PBS, pH 7.4).

As shown in Figure 4, PDI of both polymer samples increased with time. PDI for PLGA50/50 increased from week 2, but dropped to a lower value (lower than its initial PDI) on day 42, which shows it is fully degraded. PDI for PLC70/30 increased slightly in the first 56 days of study, but increased suddenly to 5 or more at the end of the study (not fully degraded at the end of the study period).

#### Thickness change with degradation

**Figure 5:**
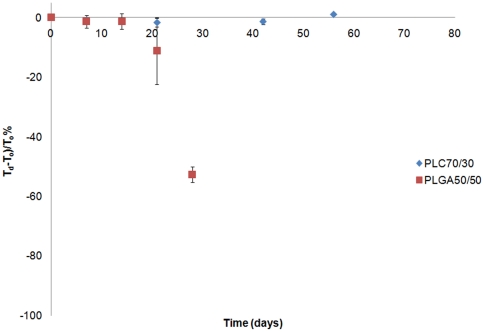
Change of film thickness of PLGA50/50 and PLC70/30 in vitro (PBS, pH 7.4).

The thickness of PLGA50/50 was unchanged (within 10%) in the first 21 days, but a sudden drop of 60% occurred on the day 28, and immeasurable on day 42,. From the observed changes on mass loss, PLGA50/50 lost 90% of its initial weight on day 42, and corresponded to the change in film thickness. In contrast, PLC70/30 maintained its shape throughout the entire duration of the study period, with minimal change in film thickness (Figure 5).

### Results of in vivo study in rabbit eyes

#### Slit-lamp examination of implanted microfilms

**Figure 6:**
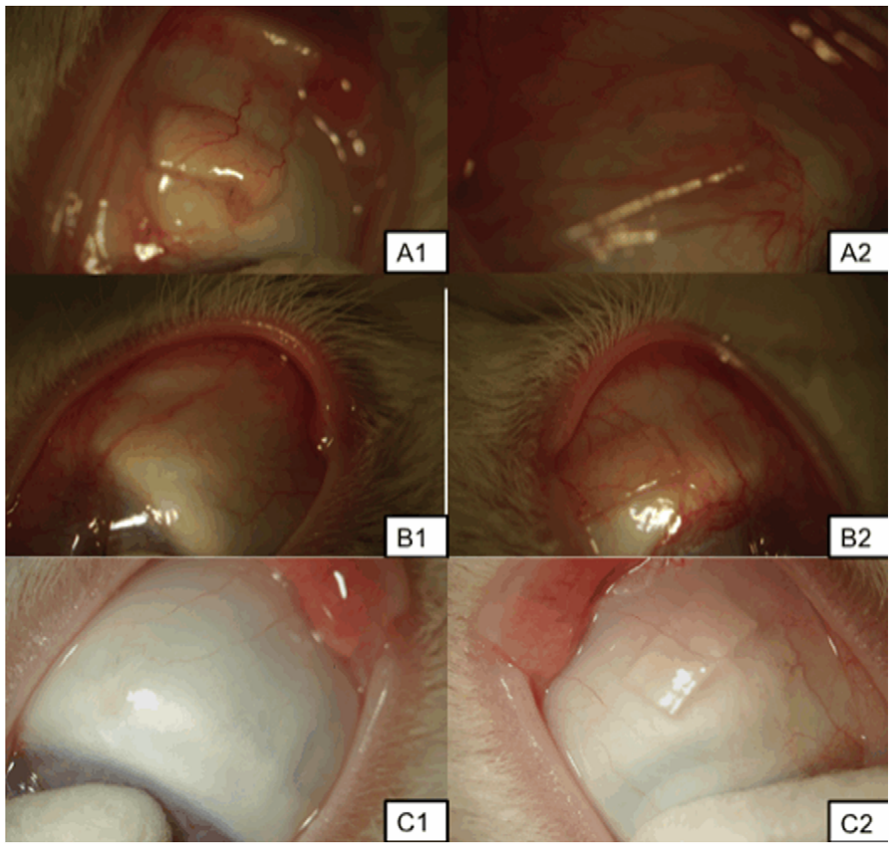
Slit-lamp photographs of microfilms after surgical insertion into the subconjunctival space at 1, 3 and 6 months. A, B, C: 1– Slit-lamp photographs of PLGA50/50 microfilm at 1, 3, 6 months respectively. A, B, C: 2 – Slit-lamp photograph of PLC70/30 microfilm at 1, 3, 6 months respectively.

The gross appearance and examination of the implanted microfilms revealed minimal localized inflammation and vascularity using serial vascularity grading scales [34] around the implanted PLC70/30 (n = 9) and PLGA50/50 (n = 9). All eyes (n = 18) had mild conjunctival hyperemia and chemosis, which resolved at one week post-operatively (Figure 6). We compared conjunctival vascularity of the insertion site before surgery and at the end of the study in all eyes. We found no significant increase in ocular score in all 18 eyes (mean percent increase in ocular score PLGA50/50 12±0.5% vs. PLC70/30 15±0.6%; *P* = 0.91, no significant inter-observer variability). The cornea, anterior chamber and lens remained clear with no evidence of inflammation or scarring. We did notice on external ocular examination that the PLGA50/50 microfilms appeared more pliable compared to the PLC70/30 microfilms, which retained its original shape. However, the animals did not display any signs of ocular discomfort, nor was there any extrusion of any of the implants from either material. At month 4 onwards, the PLGA50/50 microfilms had dissolved to such an extent that they were not grossly visible even with slit-lamp examination. In contrast, PLC70/30 microfilms persisted and visible up to 6 months.

#### Anterior Segment Optical Coherence Tomography (AS-OCT) scans

**Figure 7:**
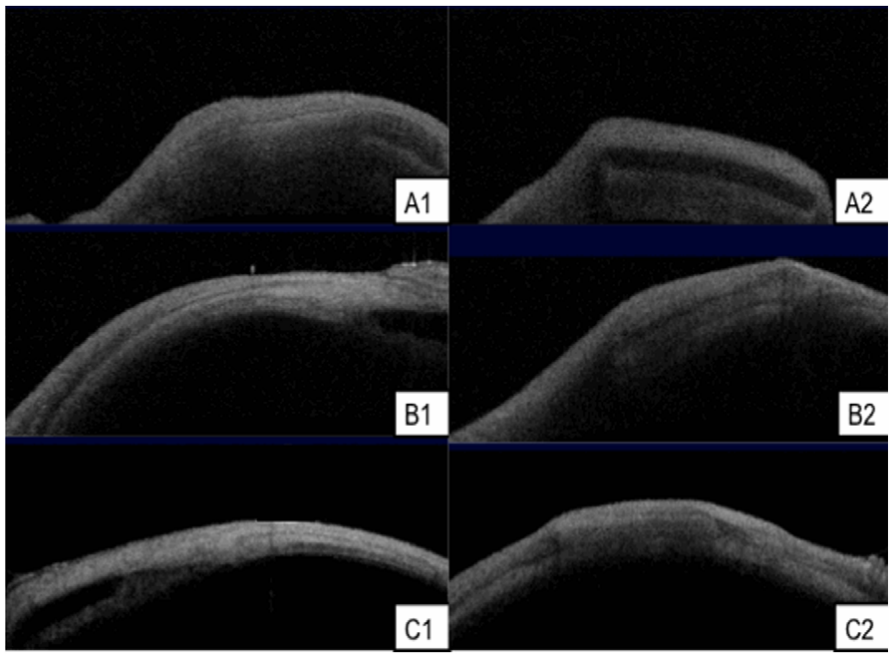
AS-OCT scans of microfilms after subconjunctival implantation. A, B, C: 1– AS-OCT of PLGA50/50 microfilms at 1, 3, 6 months respectively. A, B, C: 2 – AS-OCT of PLC70/30 microfilms at 1, 3, 6 months respectively.

**Figure 8:**
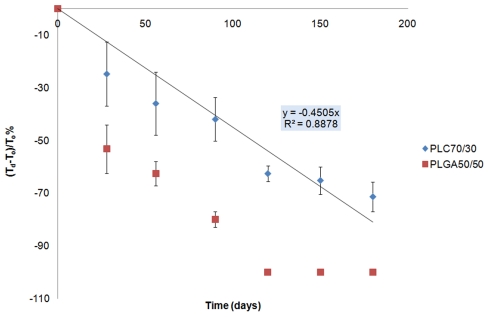
Serial AS-OCT thickness measurements of PLGA 50/50 and PLC70/30 microfilms in subconjunctival space.

The AS-OCT images taken at monthly intervals revealed good anatomical placement of all microfilms implanted (n = 18) in the subconjunctival space (Figure 7). No migration from the original surgically implanted site was seen in any of the implants. There was no evidence of scleral erosion of the subconjunctival implants in any of the eyes. Serial measurements were taken from the AS-OCT images that measured multiple sections of the implanted microfilms. We found that the thickness of both PLGA and PLC microfilms decreased, and PLC70/30 decreased in a linear fashion (R^2^ = 0.8878 for PLC70/30), as measured by AS-OCT (Figure 8).

### Histology

**Figure 9:**
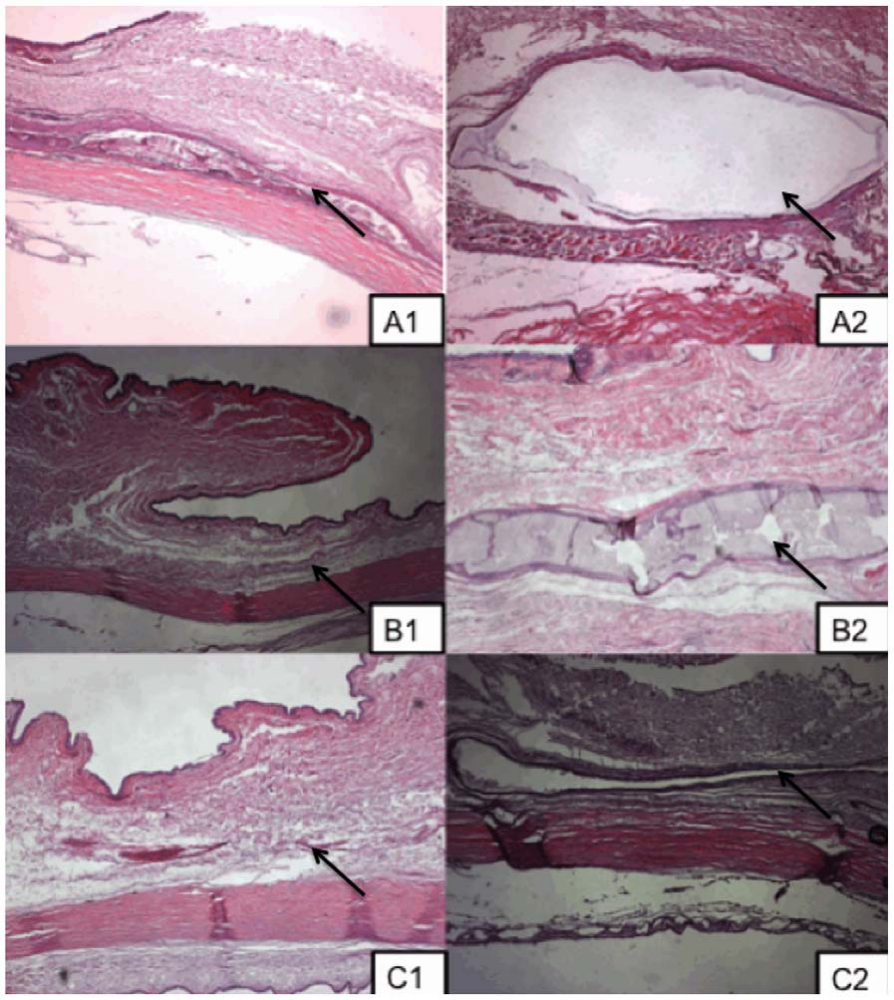
Histological sections of PLGA50/50 and PLC70/30 microfilms in subconjunctival space. A, B, C: 1– Histology of eye implanted of PLGA50/50 microfilm at 1, 3, 6 months respectively. A, B, C: 2 – Histology of eye implanted with PLC70/30 microfilm at 1, 3, 6 months respectively.

Histopathological examination of all the enucleated eyes (n = 18) revealed minimal inflammation in the initial 1–2 weeks post-operatively. This inflammation was seen to resolve by 1 month and there was minimal or no fibrosis or scarring detected. By 4 months post-implantation, the PLGA50/50 microfilms were not significantly visible on histology sections within the subconjunctival space. There was no significant fibrosis or collagen capsule formation seen around the implant site (Figure 9). However, capsule formation for PLC70/30 microfilms was noted by 3 months on the histological sections. The mean collagen capsule thickness surrounding the PLC70/30 microfilms increased from 7.5±0.026 µm at 3 months to14.75±0.11 µm at 6 months, *P*<0.001. Histological examination did not show an obvious foreign body encapsulation of the implanted films. This is important as excessive scarring and encapsulation often affects ocular function or lead to surgical failure in surgeries such as glaucoma filtration surgery [35].

### Immunohistochemistry

**Figure 10:**
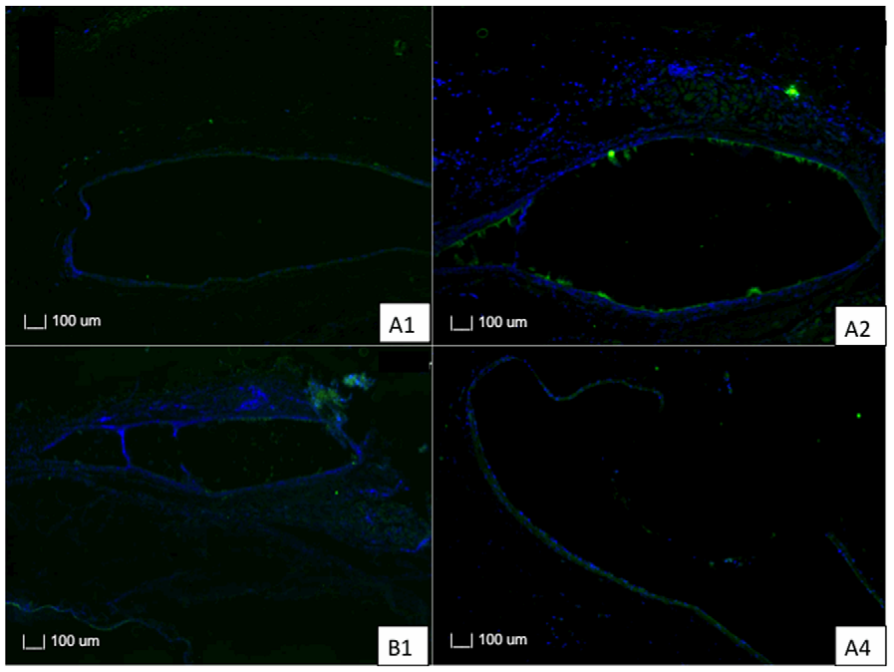
Sections of PLGA50/50 and PLC70/30 with immunohistochemistry stains for CD45 T cells. Arrows point at the implant site. A, B: 1– Section of eye implanted with PLGA50/50 microfilm at 3 and 6 months respectively. A, B: 2 – Section of eye implanted with PLC70/30 microfilm at 3 and 6 months respectively.

Sections stained with immunohistochemistry for CD45 T cells were analyzed under polarized microscopy at 3 and 6 months post-operatively. The PLC70/30 microfilms had significant reduction in amount of inflammation surrounding the capsule from 3 months post-implantation to the end of 6 months (inflammatory L/C ratio 8.5±0.6 vs. 5.5±0.8, P = 0.0018). Importantly, there was minimal infiltration of T cells detected by immunohistochemistry surrounding the implant site of the PLGA50/50 microfilms and the inflammatory L/C ratio could not be calculated (Figure 10).

### Immunofluorescence

**Figure 11:**
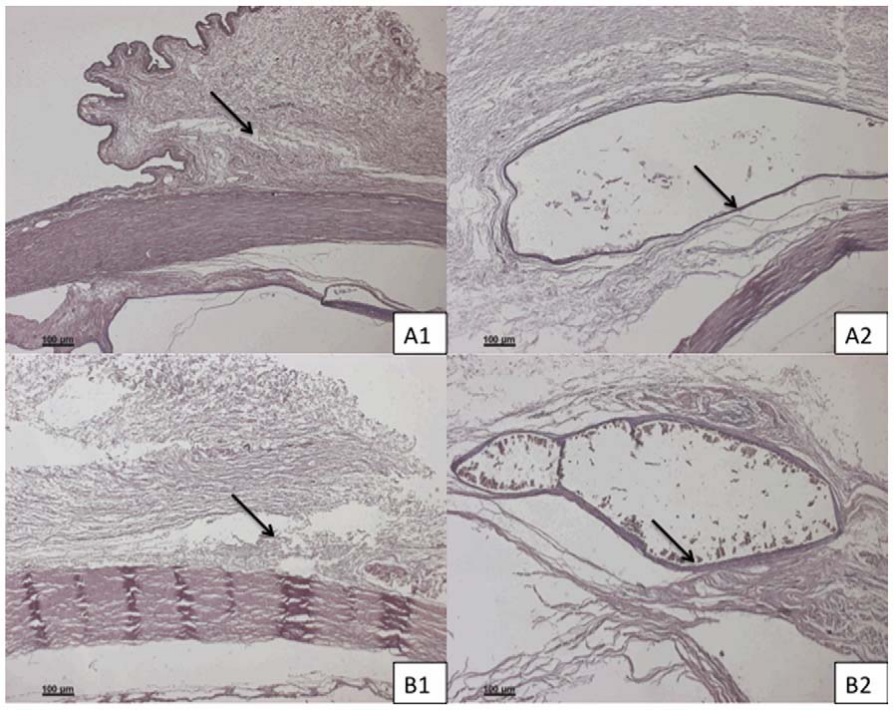
Photographs with fluorescence microscopy of sections of PLGA50/50 and PLC70/30 with immunofluorescence stains for T cells. A, B: 1– Section of eye implanted with PLGA50/50 microfilm at 3 and 6 months respectively. A, B: 2 – Section of eye implanted with PLC70/30 microfilm at 3 and 6 months respectively.

Sections stained with immunofluorescence for CD45 T cells were analyzed for both PLC70/30 and PLGA50/50 at 3 and 6 months. There was minimal inflammatory reaction, with scattered T cells surrounding both implants. We also noted a reduction in T cells surrounding the implant for both PLGA50/50 and PLC70/30 when comparing implants at 3 and 6 months post-operatively (Figure 11).

## Discussion

The current mainstay of ocular therapy is via topical administration. While it is easy to administer (for example, using eye drops), there are many drawbacks - which include poor bioavailability and penetration of the drugs, frequent instillation leading to poor compliance and blurring of vision from viscous vehicles [30]. Typically less than 5% of the topically applied drug penetrates the cornea and reaches intraocular tissues, while a major fraction of the instilled dose is often absorbed systemically via the conjunctiva and nasolacrimal duct [31]. Thus, frequent instillation of a relatively concentrated solution is required for a sustained, therapeutic effect [32]. This need for frequent instillation also leads to poor patient compliance, with often disastrous consequences for vision.

We have used biodegradable polymers to create implants that may be capable of sustained ocular drug delivery, to overcome the disadvantages of topical medications and the issues with compliance – a common problem faced by ophthalmologists in dealing with diseases such as glaucoma, the second leading cause of irreversible blindness in the world. This biopolymer microfilm placed in the subconjunctival space may significantly improve drug availability and reduce local ocular side effects, while overcoming poor patient compliance.

Our study demonstrates that both PLGA50/50 and PLC70/30 microfilms are biocompatible and safe to be inserted subconjunctivally in the rabbit eye. We also report for the first time, the use of a novel AS-OCT imaging technique to serially measure the degradation of the microfilms and describe the internal structures of the eye for encapsulation *in vivo* over a prolonged period of 6 months. We used several techniques to demonstrate the biocompatibility, as well as behavior of these microfilms when inserted into the subconjunctival space of the eye. Slit-lamp microscopy allowed us to examine the location of the microfilms, while monitoring inflammation and scarring of the overlying conjunctiva. We did not find any significant increase in inflammation or vascularity using serial vascularity grading scales [34]. We also used an imaging technique, AS-OCT, to produce cross-sectional scans of the implanted microfilms and eyes. The AS-OCT device that is commonly used in the clinic on patients rapidly captures reflections of light using low-coherence interferometry to create a cross-sectional image in 8 meridians (128 sectional maps) to produce high-resolution profile imaging of the anterior segment [28]. This enabled us to not only examine the internal structure of the eyes, but also the microfilm within the subconjunctival space in situ during the entire study period. We used the AS-OCT to serially measure the thickness of our microfilm implants with a high resolution of up to microns and monitored each implant's position. We recognize that there are potential limitations due to the resolution of the AS-OCT. However, we used a standard technique to capture the cross-sectional image of each microfilm and these cross-sectional images are taken in 8 meridians (128 sectional maps) to produce high-resolution profile. There was no obvious fibrosis, effusion and encapsulation in the neighboring ocular structures. Histological examination did not show an obvious foreign body encapsulation of the implanted films. This is important as excessive scarring and encapsulation often affects ocular function or lead to surgical failure in surgeries such as glaucoma filtration surgery [35]. All these studies revealed minimal scarring and inflammation induced by the implanted microfilm in the subconjunctiva over the 6-month study period.

It is generally accepted that, in this class of polymers used in our study (poly α-hydroxy esters), there may be two different modes of degradation. In the first mechanism, which is often referred to as homogeneous or bulk degradation, the polymers degrade slowly with no appreciable mass or volume loss until the degradation products become water-soluble and leach out of the matrix, when mass loss is then detectable. In the second mechanism, the polymer degrades first at the surface, and the surface molecules decrease in molecular weight to the point where the surface molecules leach out, without affecting the interior of the material. In this mode of degradation, which is sometimes referred to as heterogeneous degradation or surface erosion, there is continuous decrease in mass and in the material dimensions.

From the results of the study, the PLGA 50/50 films clearly exhibited bulk degradation *in vitro* and *in vivo*. Although not as evident (since no significant mass loss has been detected up to day 40 – Figure 2), *in vitro*, PLC70/30 also exhibited molecular weight decrease (Figure 3) without any mass loss, which is a characteristic of bulk degradation. However, PLC70/30 clearly behaves differently when implanted into the rabbit eyes. PLC70/30 microfilms underwent surface erosion in the subconjunctival space, as evidenced by our serial measurements using slit-lamp microscopy and AS-OCT techniques, since the width and length of the microfilms did not change visually over 6 months (Figure 6), but thickness of the films (Figure 7) decreased continuously. This is typically observed in surface erosion or heterogeneous degradation. Usually, the polymer changes from a bulk degradation mode to a surface erosion mode when the intrinsic hydrolysis rate (R_h_) becomes higher than the water ingress rate into the polymer (R_w_). We hypothesize that in the *in vivo* situation, R_h_ is being increased relative to R_w_, most likely due to the influence of enzymes (esterases) or proteins present in the eye. A surface erosion mode is the preferred mode in such applications, as bulk degradation may lead to “catastrophic” breakdown into small fragments causing localized irritation. Surface erosion also results in a constant release of incorporated drug. PLGA and PLC are anionic polymers that undergo bulk degradation *in vitro*. Embedded drugs are released from the matrix via diffusion initially, followed by degradation of the polymer matrix itself [33]. Thus the first observation of surface erosion of this grade of polymers in the sub-conjunctival space is exciting and opens the door for a more efficient therapeutic route.

The subconjunctival space is a potential area in the eye that is useful for delivering ocular drugs in a sustained manner. Currently, peribulbar or subtenon injections are used to deliver short to intermediate duration of drugs to the eye [36]. Implanting the microfilm in this space may bypass ocular blood and lymphatic barriers, to achieve therapeutic levels in the eye with lower loading concentrations of drug [37]. In this study, we have shown that the PLC70/30 and PLGA50/50 microfilms can be placed into the subconjunctival space using a simple surgical technique, and that both microfilms remain stable in-situ for up to 6 months. Furthermore, we have demonstrated that surgical implantation of these films in the subconjunctival space does not cause any associated significant scarring, encapsulation or inflammation.

In conclusion, we report that biodegradable microfilms prepared from PLGA50/50 and PLC70/30, are non-toxic and well tolerated when implanted in the subconjunctival space and therefore has the potential use as an ocular drug delivery platform. PLGA50/50 always degraded at a faster rate than PLC70/30. Both PLGA50/50 and PLC70/30 demonstrated bulk degradation *in vitro*, whereas PLC70/30 exhibited surface erosion *in vivo*. The observation of surface erosion in the sub-conjunctival space is significant for controlling the release of drugs locally, and opens the door for more efficient and sustained therapy.
